# Formulation Design and Development of a Unani Transdermal Patch for Antiemetic Therapy and Its Pharmaceutical Evaluation

**DOI:** 10.1155/2016/7602347

**Published:** 2016-06-15

**Authors:** Mohd Nauman Saleem, Mohammad Idris

**Affiliations:** ^1^Post Graduate Department of Ilm-us-Saidla, Ayurvedic & Unani Tibbia College, Karol Bagh, New Delhi 110005, India; ^2^Departments of Ilm-us-Saidla & Advia, Ayurvedic & Unani Tibbia College, Karol Bagh, New Delhi 110005, India

## Abstract

The Transdermal Drug Delivery System (TDDS) is one of the novel routes for systemic delivery of drugs through intact skin. A transdermal patch (TP) is a medicated patch that is placed on skin for delivery of medication through skin into the blood stream. The aim of present study was to formulate and evaluate a Unani transdermal patch that could be used for antiemetic therapy. The incorporation of Unani ingredients, namely, Khardal (*Brassica nigra*), Zanjabeel (*Zingiber officinale*), Podina (*Mentha arvensis*), and Sirka (Vinegar) were envisaged. The TP was prepared by solvent evaporation technique and was evaluated for organoleptic characteristics and other physicochemical properties, such as thickness, weight uniformity, folding endurance, moisture content, drug content, and tolerability and acceptability of patch. The* in vitro* permeation study of the patch was carried out through Franz diffusion cell using egg shell membrane as barrier membrane. Phosphate buffer pH 7.4 was used as dissolution medium and the temperature was maintained at 37 ± 1°C. The* in vitro* permeation study of the prepared TP indicated a time dependent increase in drug release throughout the study. The percentage of cumulative drug release was found to be 77.38% in 24 hours. The study shows a new approach to work in Unani pharmaceutics.

## 1. Introduction

The Transdermal Drug Delivery System (TDDS) is one of the novel routes for systemic delivery of drugs through the intact skin [1]. The ultimate goal of this dosage design is to maximize the flux through skin and at the same time minimize retention and metabolism of drug in the skin. It also ensures that compounds are delivered, preferably at a specific rate, to the systemic circulation [2, 3]. The TDDS can deliver certain medications to systemic circulation in a more convenient and effective way than conventional dosage form. The potential of skin as a path of drug administration has been amply demonstrated by the acceptability of marketed therapeutic systems. It is estimated that worldwide market revenues for transdermal products were around US$ 6 billion in 2009, shared between the USA (56%), Europe (32%), and Japan (7%), and are likely to reach a staggering US$ 10 billion in 2015 and even more in 2020 [4].

The TDDS offers several significant advantages such as avoidance of the hepatic-first-pass metabolism, avoidance of gastrointestinal drug absorption difficulty, and noninvasiveness. Pharmaceutically, a transdermal patch is a dosage form that is used for delivery of medication through the skin into the blood stream. Following skin permeation, the drug first reaches the systemic circulation and then is transported to the target site, which could be relatively remote from the site of administration, to produce therapeutic action [5].

Though the concept of TDDS happens to be a new one and belongs to conventional pharmacology, Unani classical literature has ample evidence of it. There are a number of single and compound Unani formulations exhibiting transdermal activity mentioned in the celebrated writings of the Unani physicians. One of the oldest evidence of delivering drugs through skin is reported in the 16th Century BC where the husk of castor oil plant in water was placed on an aching head [6]. Also the use of Khardal (*Brassica nigra*) has been in practice right from prehistoric period in the form of mustard plaster which was first described by Dioscorides (circa 1st century CE) more than two thousand years ago [7]. The mustard plasters have also been used to overcome the condition of vomiting [8, 9].

Vomiting/emesis (*qai*) is an abnormal indication of gastrointestinal tract. This situation warrants an urgent medical attention. The oral route of medication is usually not suited and parenteral therapy has various limitations and drawbacks. Thus, a need arises to explore a possible alternate route through skin, that is, transdermal.

In conventional medicine, the antiemetic treatment comes along with certain side effects. Hence, turning to safe, effective, and time-tested system of medicine, a Unani drug formulation would be a preferable option.

## 2. Objectives

The aim of the present study was to formulate and evaluate a Unani transdermal patch that could be used for antiemetic therapy.

In the present formulation, incorporation of Unani ingredients such as Khardal (*Brassica nigra*), Zanjabeel (*Zingiber officinale*), Podina (*Mentha arvensis*), and Sirka (Vinegar) was envisaged. The main ingredient of the formulation is* Khardal*, and* Sirka* is used as an excipient [10]. According to Unani literature,* Sirka* possesses* saree-ul-nufooz* (penetration enhancer) property and is also a corrective for* Khardal*.* Zanjabeel* and* Podina* have been added to the formulation as these drugs have potent antiemetic action [11–13]. At the same time these drugs are said to enhance the transdermal permeation [14, 15]. Therefore, these drugs will act as permeation enhancers, and at the same time they will produce suitable synergistic effect too.

## 3. Materials and Methods

All the ingredients of the formulation were procured from the open market. The foreign matter and impurities were inspected with naked eyes and removed. Samples of all the herbal drugs were identified by the experts of National Institute of Science Communication and Information Resources (NISCAIR), CSIR, New Delhi.

### 3.1. Extraction of Volatile Oils of* Khardal*,* Zanjabeel*, and* Podina*

For the design and development of an antiemetic formulation as a novel dosage form, firstly volatile oils from Khardal seeds, Zanjabeel rhizomes, and Podina leaves were extracted using Clevenger apparatus. The apparatus consisted of one round bottom flask of 1000 mL which was connected to the separator, in which the oil was automatically separated from the distillate in a graduated tube, thereby permitting a direct reading of the quantity of oil collected. This separator was connected to a condenser. The heat during the oil collection was kept at moderate temperature (50–70°C) and was adjusted during the course of experiment [16].

### 3.2. Preparation of Calibration Curve for* Khardal* Oil

A standard curve was prepared by dilution method. A stock solution of 10 *μ*L in 10 mL of methanol was used for making different concentrations of 1, 2, 3, 4, and 5 *μ*L/mL, respectively. The absorbances of these solutions were determined spectrophotometrically at *λ*max of 304 nm [17].

### 3.3. Preparation and Optimization of Emulsion

An oil-in-water (o/w) emulsion was prepared using tween 80 as surfactant and ethanol as cosurfactant. The oil phase was prepared by taking 75% of* Khardal* oil and 25% of mixture of equal parts of* Zanjabeel* oil and* Podina* oil. The aqueous phase used was 5% solution of* Sirka*. Emulsions were prepared in different ratios of oil phase : surfactant : aqueous phase (O : S : A) and kept at room temperature for one month to check their stability. After optimization, the most stable emulsion was selected for dosage form development [17].

### 3.4. Formulation of an Antiemetic Unani Transdermal Patch

The patch was prepared by solvent evaporation technique. Initially, 4% solution of water and ethanol mixture (taken in the ratio 1 : 1) was prepared. Then 4% lactic acid solution was prepared using the previously prepared solution. Then 5 mL of this solution was taken and the temperature of this solution was maintained at 37 ± 1°C using a hot plate. Later on, 125 mg of chitosan was slowly and gradually added into it and dissolved using magnetic stirrer. After the complete dissolution of chitosan, 1 mL of PEG-400 (Polyethylene glycol) was added which was followed by addition of 1 mL of distilled water. The mixture was stirred well to obtain a thick uniform solution. Then 1 mL of previously optimized emulsion was added dropwise and stirred well. The solution was then poured into the mould having an area of 4 × 2 cm^2^ and left overnight for drying at room temperature. After drying, two patches of size 2 × 2 cm^2^ were obtained [18].

### 3.5. Evaluation of Prepared Patches

#### 3.5.1. Organoleptic Characteristics

The prepared patch was physically inspected for its appearance, colour, clarity, flexibility, and smoothness.

#### 3.5.2. Thickness

The thickness of patch was measured by Vernier calipers. The thickness uniformity was measured at different sites and average was calculated [19].

#### 3.5.3. Weight Uniformity

Three patches of equal size were taken and weighed on electronic balance to check for weight variation [19].

#### 3.5.4. Folding Endurance

The patch was taken and folded repeatedly at same point till it breaks. The number of times patch could be folded without breaking was noted [20].

#### 3.5.5. Moisture Content

The prepared patch was weighed and kept in the dessicator containing fused calcium chloride for about 24 hours. After that it was taken out and weighed again [20]. The percentage of moisture content was calculated on the basis of the following formula:

(1) Percentage  of  moisture  content=Initial  weight−Final  weightFinal  weight×100.

#### 3.5.6. Drug Content

The patch was dissolved in methanol and the remaining volume was made up with distilled water to 100 mL. Then the solution was filtered and the absorbance of the solution was taken at 304 nm and concentration was calculated [21].

#### 3.5.7. *In Vitro* Permeation Study of Patch

The* in vitro* permeation study of the prepared patch was carried out through egg shell membrane because the egg shell membrane resembles human stratum corneum as it consists mainly of keratin [22]. The membrane was accordingly prepared before use [23]. The water in the outer jacket of the cell was warmed and set at 37 ± 1°C throughout the experiments to provide a skin surface temperature. Phosphate buffer solution of pH 7.4 was used as dissolution medium in the receptor compartment. A 5 × 5 mm^2^ piece of patch was taken and applied over the mounted membrane in diffusion cell. After that, the samples were withdrawn from the receptor compartment at regulated intervals. The sampling schedule was at 0, 15, 30, and 60 minutes for the first hour of release and then it was at every hour interval till 6th hour of release. After that the whole system was kept in its normal position overnight and then next day reading was taken at 24th hour. One mL of the receptor solution was collected as sample each time and simultaneously one mL of phosphate buffer solution was added back to the receptor cell for maintaining the same initial volume of the receptor cell solution. The collected samples were analysed using UV-Vis spectrophotometer [19].

## 4. Results and Discussion

Vomiting/emesis is an emergent situation for which oral route of medication is usually not suited, and the medication is provided* en route* parenterally which is not available in Unani system of medicine. However, there are a number of antiemetic formulations mentioned in the celebrated writings of the Unani physicians which are topically applied over the skin to achieve desired therapeutic action. So, a pharmaceutical strategy was envisaged to generate significant scientific data by designing and developing a novel, safe, noninvasive, and patient-friendly dosage form, that is, transdermal drug delivery dosage form.

For the design and development of antiemetic formulation into novel dosage form, firstly volatile oils from different ingredients were extracted. The rationale behind extraction of volatile oils is that according to literature volatile oil form is best suited for transdermal drug delivery as it can penetrate more through the skin and the dosage is also reduced. Also in this case volatile oil contained the active constituents of the respective drugs.

**Figure 1:**
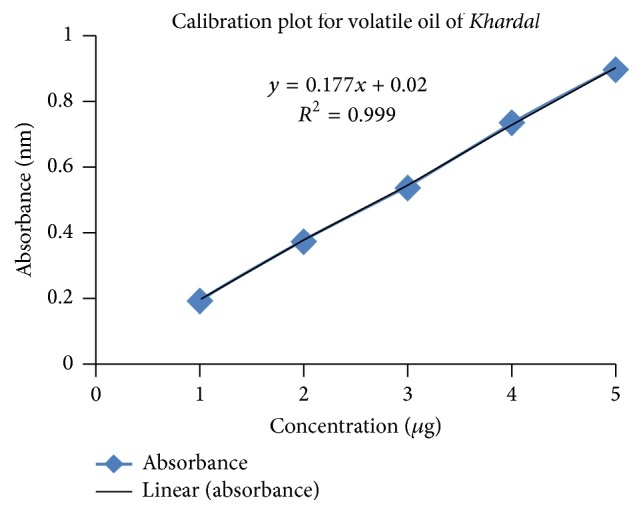
Calibration curve of volatile oil of* Khardal*.

Initially a standard calibration curve for* Khardal* oil was prepared. The absorbance values are given in Table 1. Using concentration and absorbance data, Beer and Lambert's plot was obtained. The plot is shown in Figure 1.

### 4.1. Organoleptic Characteristics

**Figure 2:**
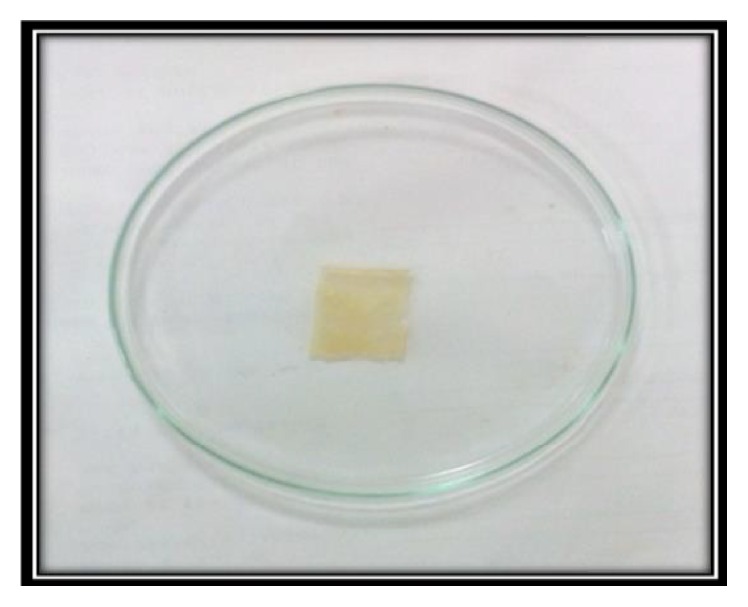
Sample of prepared Unani transdermal antiemetic patch.

The prepared patches were slightly opaque, pale coloured, jellified preparations showing good flexibility and smoothness as given in Table 2. A sample of prepared patch is shown in Figure 2.

### 4.2. Thickness

The mean thickness of prepared patches was 0.6 ± 0.026 mm as given in Table 3.

### 4.3. Weight Uniformity

The mean weight of prepared patches was 0.251 ± 0.005 gm as given in Table 4.

### 4.4. Folding Endurance

The mean folding endurance of prepared patches was 77.33 ± 2.081 as given in Table 5.

### 4.5. Moisture Content

The mean moisture content of prepared patches was 4.91 ± 0.184% as given in Table 6.

### 4.6. Drug Content

The mean drug content of the prepared patches was 0.284 ± 0.007 mg in w/w ratio with the weight of patch as given in Table 7.

### 4.7. *In Vitro* Permeation Study of Patch

**Figure 3:**
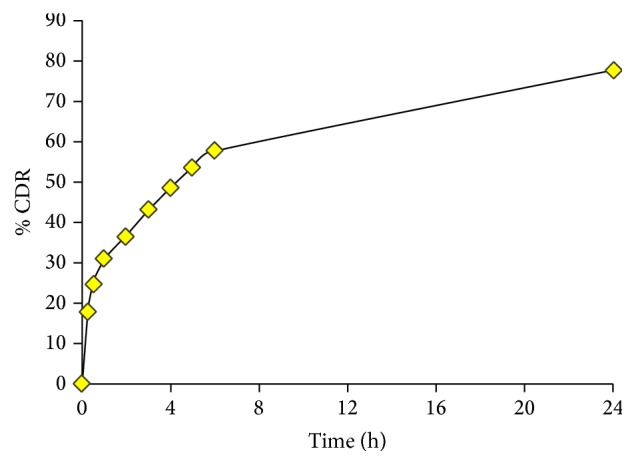
*In vitro* permeation study of patch.

The* in vitro* permeation studies of the prepared transdermal patches indicated a time dependent increase throughout the study as shown in Figure 3. The drug release from patches was rapid during first hour and it slowed down thereafter. The percentage of drug release was 17.83% in 15 minutes which further increased to 24.56% in 30 minutes and reached 30.89% in one hour. The cumulative drug release increased gradually and reached 57.91% in 6 hours. Finally, at the end of study, the cumulative drug release reached a remarkable peak, that is, 77.38% in 24 hours, as given in Table 8.

## 5. Conclusion

Keeping in view the current Unani pharmaceutical scenario, it is the felt-need of the hour to envisage a research study on design and development of Unani dosage forms, since there is no parenteral dosage form available in Unani system of medicine. Accordingly, the study was based on design and development of an antiemetic transdermal Unani formulation in a novel dosage form for a common clinical condition, namely, vomiting/emesis (*qai*). The patch was found to be stable and showed no signs of skin irritation. The study shows a new approach to work in Unani pharmaceutics.

Further, a clinical study is warranted to evaluate therapeutic efficacy of this novel dosage form.

## Figures and Tables

**Table 1:** Standard calibration data for volatile oil of *Khardal*.

Serial number	Concentration (*µ*g)	Absorbance (nm)
(1)	15.625	0.077
(2)	31.25	0.112
(3)	62.5	0.171
(4)	125	0.285
(5)	250	0.501
(6)	500	0.932

**Table 2:** Organoleptic characteristics of the prepared patches.

Serial number	Physical characteristic	Result
(1)	Appearance	Jellified preparation
(2)	Colour	Pale
(3)	Clarity	Slightly opaque
(4)	Flexibility	Good
(5)	Smoothness	Good

**Table 3:** Thickness of patch.

Serial number	Thickness (mm)
(1)	0.63
(2)	0.58
(3)	0.59

Mean ± SD	0.6 ± 0.026

**Table 4:** Weight uniformity of patch.

Serial number	Weight (gm)
(1)	0.257
(2)	0.249
(3)	0.248

Mean ± SD	0.251 ± 0.005

**Table 5:** Folding endurance of patch.

Serial number	Folding endurance
(1)	75
(2)	78
(3)	79

Mean ± SD	77.33 ± 2.081

**Table 6:** Moisture content of patch.

Serial number	Moisture content (%)
(1)	5.07
(2)	4.71
(3)	4.96

Mean ± SD	4.91 ± 0.184

**Table 7:** Drug content of patch.

Serial number	Drug content (mg)
(1)	0.292
(2)	0.281
(3)	0.278

Mean ± SD	0.284 ± 0.007

**Table 8:** *In vitro* permeation study of patch.

Serial number	Time of collection	Concentration (*µ*L/mL)	% CDR
(1)	0 minutes	0	0
(2)	15 minutes	6.686	17.83
(3)	30 minutes	9.210	24.56
(4)	1 hour	11.584	30.89
(5)	2 hours	13.699	36.53
(6)	3 hours	16.136	43.03
(7)	4 hours	18.233	48.62
(8)	5 hours	20.051	53.47
(9)	6 hours	21.716	57.91
(10)	24 hours	29.018	77.38
